# Real-World Persistence of Successive Biologics in Patients With Inflammatory Bowel Disease: Findings From ROTARY

**DOI:** 10.1093/ibd/izad245

**Published:** 2023-10-31

**Authors:** Noa Krugliak Cleveland, Sabyasachi Ghosh, Benjamin Chastek, Tim Bancroft, Ninfa Candela, Tao Fan, Kandavadivu Umashankar, David T Rubin

**Affiliations:** Inflammatory Bowel Disease Center, University of Chicago Medicine, Chicago, IL, USA; Takeda Pharmaceuticals USA, Inc, Lexington, MA, USA; Optum, Eden Prairie, MN, USA; Optum, Eden Prairie, MN, USA; Takeda Pharmaceuticals USA, Inc, Lexington, MA, USA; Takeda Pharmaceuticals USA, Inc, Lexington, MA, USA; Takeda Pharmaceuticals USA, Inc, Lexington, MA, USA; Inflammatory Bowel Disease Center, University of Chicago Medicine, Chicago, IL, USA

**Keywords:** inflammatory bowel disease, biologics, sequencing, Crohn’s disease, ulcerative colitis

## Abstract

**Background:**

Patients with inflammatory bowel disease (IBD) may receive multiple successive biologic treatments in clinical practice; however, data are limited on the comparative effectiveness of biologics and the impact of treatment sequence on outcomes.

**Methods:**

The ROTARY (Real wOrld ouTcomes Across tReatment sequences in inflammatorY bowel disease patients) study was a retrospective, observational cohort study conducted using data from the Optum Clinical Database between January 1, 2012, and February 29, 2020. Adult patients with Crohn’s disease (CD) or ulcerative colitis (UC) who received 2 biologics successively were included. Biologic treatment sequences were analyzed descriptively. Cox proportional hazards models, adjusted for baseline demographics and clinical characteristics, were used to estimate the hazard ratio of switching or discontinuation for each first- and second-line biologic compared with first- and second-line adalimumab, respectively.

**Results:**

In total, 4648 patients with IBD (CD, n = 3008; UC, n = 1640) were identified. Most patients received tumor necrosis factor α antagonist (anti-TNFα) treatment followed by another anti-TNFα treatment or vedolizumab. Vedolizumab and infliximab had 39.4% and 34.6% lower rates of switching or discontinuation than adalimumab, respectively, as first-line biologics in patients with CD and 30.8% and 34.3% lower rates as first-line biologics in patients with UC, respectively. Vedolizumab, infliximab, and ustekinumab had 47.2%, 40.0%, and 43.5% lower rates of switching or discontinuation than adalimumab, respectively, as second-line biologics in CD and 56.5%, 43.0%, and 45.6% lower rates as second-line biologics in patients with UC, respectively.

**Conclusions:**

Although anti-TNFα treatments were most commonly prescribed, the adjusted rates of discontinuation for adalimumab as both a first- and second-line biologic were higher than for vedolizumab, infliximab, or ustekinumab.

## Graphical Abstract

**Graphical Abstract:**
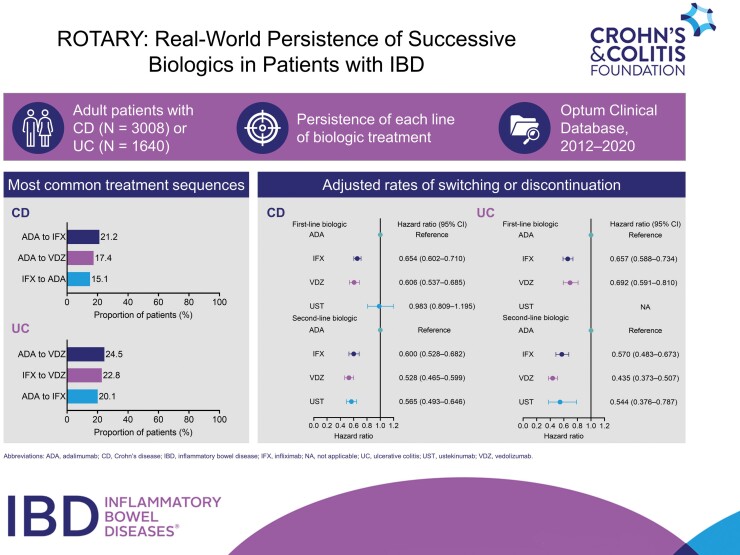

**Key Messages**

What is already known?

- Patients with inflammatory bowel disease (IBD) may require multiple successive lines of biologics to induce and maintain remission.
- The choice of biologic may affect the efficacy of subsequent lines of biologics with different mechanisms of action.

What is new here?

- Most patients with IBD who receive biologics receive a tumor necrosis factor α antagonist treatment followed by another tumor necrosis factor α antagonist treatment or vedolizumab.
- Vedolizumab and infliximab have better persistence than adalimumab as first- or second-line biologics in patients with IBD receiving 2 successive biologics.
- Ustekinumab has better persistence than adalimumab as a second-line biologic in patients with IBD receiving 2 successive biologics.

How can this study help patient care?

- A better understanding of treatment switching and discontinuation in patients with IBD could help to inform treatment decisions and improve treatment persistence in clinical practice, which may have a positive impact on rates of remission.

## Introduction

Inflammatory bowel disease (IBD) encompasses ulcerative colitis (UC), a chronic disease characterized by inflammation of the mucosa of the colon and rectum,^1-3^ and Crohn’s disease (CD), a chronic inflammatory condition that affects the entire gastrointestinal tract.^4,5^ Both UC and CD are progressive and disabling disorders with a relapsing and remitting clinical course.^1-5^

Several biologics with different mechanisms of action are available for the induction and maintenance of remission in patients with moderately to severely active CD and UC, including the tumor necrosis factor α antagonist (anti-TNFα) treatments adalimumab and infliximab, the anti-interleukin-12/23 ustekinumab, the gut-selective anti-α4β7-integrin vedolizumab, and the anti-interleukin-23 risankizumab, which was recently approved for CD.^3,5,6^ The advent of biologics was a significant breakthrough for IBD, resulting in more treatment options and better patient outcomes.^7^ However, in clinical practice, patients may discontinue their initial biologic treatment owing to nonresponse, loss of response, or intolerance, and subsequently require a second-line biologic to achieve and maintain remission.^8^ Therefore, the choice of initial biologic and subsequent lines of biologic treatment is important because it may impact the efficacy of treatment. Indeed, rates of clinical remission and endoscopic improvement are greater in patients with UC receiving adalimumab or vedolizumab who have not p?reviously been treated with an anti-TNFα than in those who have; however, data to guide biologic treatment sequencing are limited.^9^

Clinical guidelines provide treatment recommendations for biologic-naive patients with IBD and for those who have experienced nonresponse or loss of response to previous lines of biologics^10-13^; however, these are constrained by limited availability of data on the comparative effectiveness of biologics and the impact of treatment sequence on outcomes.^14^ Here, we report the real-world biologic treatment sequences received by patients with IBD and the persistence of each biologic treatment line.

## Methods

### Objectives

The aim of the ROTARY (Real wOrld ouTcomes Across tReatment sequences in inflammatorY bowel disease patients) study was to describe the sequence of biologic treatments received by patients with CD or UC in clinical practice and compare outcomes on the first 2 lines of biologic treatments.

### Study Design

The ROTARY study was a retrospective, observational cohort study of patients with IBD treated with 2 biologic treatments successively, conducted using electronic health record (EHR) data from the Optum Clinical Database between January 1, 2012, and February 29, 2020 (Supplementary Figure 1).

### Data Source

The Optum Clinical Database aggregates data from clinical encounters from over 140 000 healthcare providers in the United States. The database contains extensive de-identified patient data, including demographics, medications prescribed and administered, immunizations, allergies, vital signs and other observable measurements, administrative data relating to clinical and inpatient visits, and coded diagnoses and procedures. These data provide a longitudinal view of patients’ medical history with minimal missing data and loss to follow-up.

### Study Population

Patients with ≥1 prescription or administration of adalimumab, infliximab, vedolizumab, or ustekinumab during the patient identification period (January 1, 2013, to February 29, 2020) were included, with the date of first prescription or administration defined as the index date. Eligible patients were required to have only 1 qualifying biologic treatment on the index date, have a minimum of 12 months’ EHR activity before the index date, be ≥18 years of age on the index date with valid demographic information, and have ≥2 diagnoses of either CD or UC during the baseline period and ≥1 additional diagnosis consistent with the baseline diagnosis during follow-up, identified using International Classification of Diseases–Ninth Revision or International Classification of Diseases–Tenth Revision codes. Patients were also required to have ≥1 prescription or administration of adalimumab, infliximab, vedolizumab, or ustekinumab following the first line of biologic treatment and be treated with only 1 biologic during the second line of treatment. Patients were excluded for prescription or administration of adalimumab, infliximab, vedolizumab, or ustekinumab during the baseline period, or a diagnosis of rheumatoid arthritis, psoriatic arthritis, ankylosing spondylitis, plaque psoriasis, hidradenitis suppurativa, or noninfectious uveitis in the 6 months before the index date.

Patients were assigned to the CD or UC cohort based on their diagnoses during the baseline period. To minimize misclassification bias, patients with diagnoses for both CD and UC required ≥3 consecutive CD diagnoses following a UC diagnosis for inclusion in the CD cohort or ≥3 consecutive UC diagnoses following a CD diagnosis for inclusion in the UC cohort.

The study was conducted in accordance with the protocol, the Declaration of Helsinki, and the Good Pharmacoepidemiology Practices guidelines.

### Variables

Baseline variables were captured using data recorded on the index date or the closest date to the index date, or over the entire baseline period (ie, the 12-month period before, but not including, the index date), depending on the variable. Demographics included age, sex, race/ethnicity, and insurance type. Clinical characteristics included Charlson Comorbidity Index (CCI) score, comorbid conditions, disease extent for UC and disease location for CD, disease characteristics (CD only), smoking status, body mass index, all-cause hospitalization, duration of conventional therapy (including aminosalicylates, corticosteroids, or immunomodulating therapy), and extraintestinal manifestations.

### Endpoints

Treatment sequences were identified by the first- and second-line biologic treatments prescribed or administered during follow-up, which was defined as the period between the index date and whichever came first: the end of the second line of biologic treatment or the end of the study period. Medication administration was identified from medication administration and procedure fields in the EHR utilizing National Drug Codes and Healthcare Common Procedure Coding System codes related to each medication. For prescription orders, the date of the order, number of refills, and days of supply were used to impute the runout date using the formula: *runout date* = *date of prescription order* + [(*number of refills* + 1) × *days of supply*].

The primary endpoint was persistence on therapy, defined as the time from initiation of the qualifying biologic until whichever came first of switching, discontinuation, or the end of the study period. Switching was defined as initiation of a new qualifying biologic, with the date of switching defined as the date of prescription or administration of the new biologic. Discontinuation was defined as a treatment gap of ≥60 days for adalimumab and ≥120 days for infliximab, vedolizumab, and ustekinumab.

### Statistical Analysis

The CD and UC cohorts were analyzed separately. The demographics and clinical characteristics of patients were described overall and stratified by treatment sequence. For continuous variables, means, standard deviations, medians, ranges, and percentiles were calculated, as appropriate, or the variable was categorized. The number and proportion of patients were recorded for categorical variables. Kaplan-Meier analysis of time to switching or discontinuation was conducted for each line of biologic treatment. Cox proportional hazards models were used to estimate the hazard ratio (HR) of switching or discontinuation for each first- and second-line biologic compared with first- and second-line adalimumab, respectively. Analyses were adjusted for potential confounders, which included first- or second-line biologics (depending on the model), age, sex, race/ethnicity, body mass index, baseline smoking status, baseline CD-related conditions (for the CD cohort), baseline disease location (for the CD cohort), baseline extraintestinal manifestations (for the CD cohort), baseline disease extent (for the UC cohort), baseline all-cause hospitalization, baseline CCI score, baseline mental disorder, and baseline duration of conventional therapy, as well as the year of index date. In addition, Cox proportional hazards models were used to estimate the HR of switching or discontinuation for each individual treatment as first- or second-line biologics. Statistical analyses were performed as exploratory analyses with no a priori hypotheses using SAS v9.4 (SAS Institute) or later. For all comparisons, a significance level of .05 on a 2-tailed test was used to determine statistical significance. For comparisons among treatment sequences, *P* values were adjusted using the Bonferroni correction method.

## Results

### Patient Attrition

A total of 13 641 patients with CD and 7109 patients with UC received an index biologic. Of these patients, 22.1% of patients with CD (n = 3008) and 23.1% of patients with UC (n = 1640) subsequently received a second line of biologic treatment and met all eligibility criteria (Table 1; Supplementary Tables 1 and 2).

**Table 1.** Patients who received a second-line biologic among index biologic users.

Index biologic	Total^a^	1 line only	2 lines or more^b^	
			n	%
Crohn’s disease				
Overall	13 641	10 633	3008	22.1
Adalimumab	6756	5151	1605	23.8
Infliximab	4266	3310	956	22.4
Vedolizumab	1647	1318	329	20.0
Ustekinumab	972	854	118	12.1
Ulcerative colitis				
Overall	7109	5469	1640^c^	23.1
Adalimumab	2902	2138	764	26.3
Infliximab	2780	2119	661	23.8
Vedolizumab	1378	1165	213	15.5

^a^Excluding patients who received ≥2 lines of therapy who received >1 biologic during the second line.

^b^Patients who received ≥2 lines of treatment and met all selection criteria.

^c^Including 2 patients with ulcerative colitis who received ustekinumab as a first-line biologic.

### Treatment Sequences

**Figure 1.**
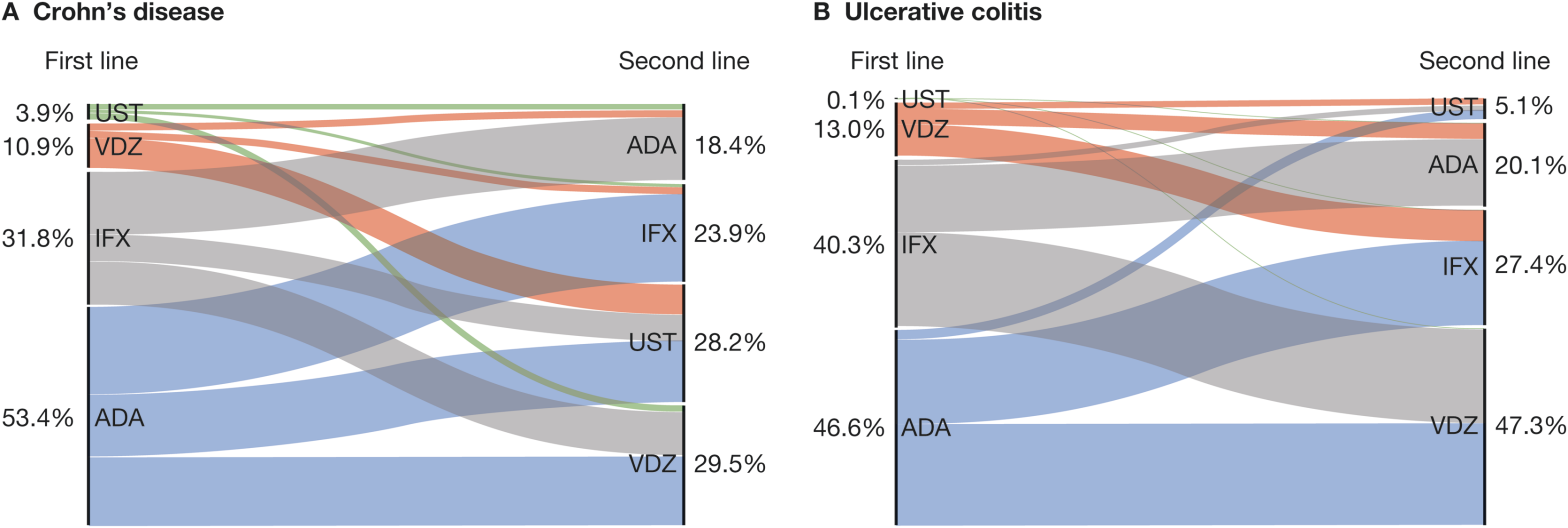
Biologic treatment sequences in patients with (A) Crohn's disease and (B) ulcerative colitis. ADA, adalimumab, IFX, infliximab, UST, ustekinumab; VDZ, vedolizumab.

Among patients with CD, the most common treatment sequences were adalimumab to infliximab (n = 637 [21.2%]), adalimumab to vedolizumab (n = 522 [17.4%]), and infliximab to adalimumab (n = 454 [15.1%]). The most common treatment sequences for patients with UC were adalimumab to vedolizumab (n = 401 [24.5%]), infliximab to vedolizumab (n = 374 [22.8%]), and adalimumab to infliximab (n = 330 [20.1%]). The proportion of patients with CD and UC receiving each line of biologic treatment is shown in Figure 1A and 1B, respectively. As only 2 (0.1%) patients with UC received ustekinumab as a first-line biologic, this line of treatment was excluded from subsequent analyses.

### Baseline Demographics and Clinical Characteristics

The mean age of patients in the overall CD cohort was 41.9 years. Most patients were female (n = 1763 [58.6%]), were White or Caucasian (n = 2679 [89.1%]), and had commercial insurance coverage (n = 1880 [62.5%]) (Table 2). The mean CCI score of patients was 0.6; mental disorders and cardiovascular disease were the most common comorbidities, affecting 25.6% (n = 769) and 23.9% (n = 718) of patients, respectively. Overall, 36.7% (n = 1105) of patients had ileocolonic disease, 24.3% (n = 730) had colonic disease, 19.1% (n = 574) had ileal disease, and 19.9% (n = 599) had unspecified disease. With regard to disease characteristics, 11.6% (n = 350) of patients had fistulas, 4.7% (n = 141) had perianal disease, 4.7% (n = 141) had abscesses, and 0.2% (n = 7) had strictures. The demographics and clinical characteristics were generally similar between patients with CD across the different treatment sequences, although a greater proportion of patients who received ustekinumab as a first-line biologic had ileocolonic disease than patients who received adalimumab, vedolizumab, or infliximab as a first-line biologic.

**Table 2.** Baseline demographic and clinical characteristics stratified by treatment sequence in patients with Crohn’s disease.

	Total (N = 3008)	ADA to IFX (n = 637)	ADA to VDZ (n = 522)	ADA to UST (n = 446)	IFX to ADA (n = 454)	IFX to VDZ (n = 317)	IFX to UST (n = 185)	VDZ to ADA (n = 55)	VDZ to IFX (n = 56)	VDZ to UST (n = 218)	UST to ADA (n = 44)	UST to IFX (n = 27)	UST to VDZ (n = 47)
Demographic													
Age, y	41.9 (15.3)	40.6 (15.3)	43.3 (15.2)	42.0 (14.1)	39.9 (14.7)	44.8 (17.4)	39.5 (15.2)	44.7 (13.4)	40.8 (14.8)	44.2 (15.7)	40.8 (14.9)	43.2 (15.5)	39.3 (15.1)
Sex													
Female	58.6	58.7	62.8	57.6	54.4	56.8	57.8	61.8	58.9	64.2	52.3	48.2	57.5
Male	41.4	41.3	37.2	42.4	45.6	43.2	42.2	38.2	41.1	35.8	47.7	51.9	42.6
Race/ethnicity													
Asian	0.8	0.6	0.4	0.7	0.7	1.6	0.5	0.0	0.0	1.4	0.0	3.7	6.4
Black or African American	7.2	8.0	5.8	4.3	9.7	7.9	6.5	5.5	5.4	8.3	6.8	22.2	4.3
White or Caucasian	89.1	88.4	91.2	92.8	86.1	87.1	88.7	90.9	94.6	87.6	90.9	70.4	89.4
Unknown or other	2.9	3.0	2.7	2.2	3.5	3.5	4.3	3.6	0.0	2.8	2.3	3.7	0.0
Insurance type													
Commercial	62.5	60.0	66.7	66.4	58.8	56.8	62.7	61.8	71.4	61.5	68.2	55.6	80.9
Medicaid	9.0	10.5	6.5	4.9	12.1	10.7	12.4	10.9	12.5	5.5	11.4	18.5	0.0
Medicare	7.6	7.9	7.1	4.3	5.3	12.6	7.0	5.5	3.6	15.6	2.3	7.4	8.5
Commercial/Medicaid	3.3	4.7	2.7	2.0	4.4	2.5	3.2	5.5	1.8	2.8	0.0	0.0	2.1
Commercial/Medicare	4.1	3.6	4.0	3.8	2.9	5.7	5.4	3.6	5.4	5.1	2.3	11.1	2.1
Commercial/Medicaid/ Medicare	0.2	0.5	0.0	0.0	0.2	0.0	0.5	0.0	0.0	0.0	0.0	3.7	0.0
Uninsured	1.1	0.6	0.4	2.2	2.2	2.5	0.0	0.0	0.0	0.0	0.0	0.0	0.0
Missing, unknown, or other	10.9	10.8	11.3	15.7	12.1	7.3	6.0	12.7	3.6	9.2	15.9	3.7	6.4
Clinical characteristics													
Conditions in the pre–index period^a^													
Mental disorder	25.6	26.1	27.6	22.0	23.6	27.1	27.6	16.4	30.4	29.4	25.0	18.5	23.4
Cardiovascular disease	23.9	24.0	22.0	22.9	21.4	28.1	23.8	25.5	21.4	29.8	22.7	11.1	29.8
Chronic pulmonary disease	15.1	16.3	15.1	11.7	16.5	17.7	12.4	20.0	7.1	14.2	13.6	14.8	19.2
Liver disease	7.0	6.0	8.1	6.1	6.8	5.4	8.1	10.9	10.7	9.2	6.8	3.7	8.5
Diabetes mellitus	5.9	7.5	6.7	5.8	4.9	6.6	3.2	1.8	1.8	6.0	0.0	0.0	6.4
CCI score	0.6 (1.1)	0.5 (1.0)	0.6 (1.1)	0.6 (1.1)	0.5 (1.0)	0.6 (1.4)	0.6 (1.1)	0.8 (1.2)	0.6 (0.9)	0.8 (1.3)	0.8 (1.3)	0.5 (0.9)	0.7 (1.3)
Body mass index, kg/m^2^	27.1 (6.8)	27.4 (6.8)	27.9 (7.1)	27.4 (6.6)	26.6 (6.8)	27.1 (7.0)	26.3 (6.4)	26.4 (6.4)	24.7 (5.5)	26.3 (6.3)	25.9 (5.5)	26.2 (7.5)	26.5 (7.0)
Smoking	21.0	23.4	19.7	17.5	23.8	21.5	24.3	14.6	21.4	17.0	29.6	11.1	19.2
Disease characteristics													
Perianal disease	4.7	4.1	2.9	3.4	6.4	4.7	10.8	0.0	3.6	6.0	4.6	0.0	8.5
Fistula	11.6	12.1	9.2	8.7	15.0	11.0	15.1	3.6	10.7	14.7	13.6	11.1	12.8
Abscess	4.7	4.1	2.9	3.4	6.4	4.7	10.8	0.0	3.6	6.0	4.6	0.0	8.5
Stricture	0.2	0.2	0.6	0.0	0.4	0.0	0.0	0.0	0.0	0.5	0.0	0.0	0.0
Disease location													
Ileum-colon	36.7	33.8	33.1	37.2	34.1	33.8	37.3	38.2	33.9	50.9	63.6	51.9	57.5
Colon	24.3	24.7	24.5	22.4	26.0	31.2	25.4	29.1	28.6	15.1	11.4	11.1	17.0
Ileum	19.1	21.0	19.5	19.7	18.9	16.7	15.1	16.4	19.6	17.9	20.5	25.9	17.0
Unspecified	19.9	20.6	22.8	20.6	20.9	18.3	22.2	16.4	17.9	16.1	4.6	11.1	8.5
Duration of conventional therapy, days													
0	26.6	23.1	28.9	28.3	22.0	28.7	36.8	25.5	23.2	26.2	25.0	25.9	29.8
1-30	11.3	12.1	9.8	9.6	13.4	10.7	14.6	0.0	16.1	8.7	20.5	7.4	14.9
31-60	13.6	12.9	11.3	11.7	18.7	13.3	14.6	14.6	10.7	12.4	22.7	22.2	8.5
61-90	9.7	9.6	8.8	10.3	9.5	8.8	10.8	20.0	10.7	8.3	6.8	7.4	19.2
≥91	38.9	42.4	41.2	40.1	36.3	38.5	23.2	40.0	39.3	44.5	25.0	37.0	27.7

Values are mean (SD) or %.

Abbreviations: ADA, adalimumab; CCI, Charlson Comorbidity Index; IFX, infliximab; UST, ustekinumab; VDZ, vedolizumab.

^a^The 5 most prevalent conditions in the total Crohn’s disease cohort.

The mean age of patients in the UC cohort was 43.7 years. Approximately half of patients (n = 812 [49.5%]) were male, and most were White or Caucasian (n = 1445 [88.1%]) and had commercial insurance coverage (n = 1080 [65.9%]) (Table 3). The mean CCI score of patients with UC was 0.5, with cardiovascular disease (n = 424 [25.9%]) and mental disorders (n = 315 [19.2%]) being the most common comorbidities. Overall, 45.2% (n = 742) of patients had pancolitis, 9.8% (n = 161) had left-sided disease, 7.9% (n = 130) had proctosigmoiditis, and 37.0% (n = 607) had proctitis, other, or unspecified disease. When stratified by treatment sequence, patients who received vedolizumab as a first-line biologic were on average older and had a slightly higher mean CCI score than patients who received adalimumab or infliximab as a first-line biologic.

**Table 3.** Baseline demographic and clinical characteristics stratified by treatment sequence in patients with ulcerative colitis.

	Total (N = 1640)^a^	ADA to IFX (n = 330)	ADA to VDZ (n = 401)	ADA to UST (n = 33)	IFX to ADA (n = 266)	IFX to VDZ (n = 374)	IFX to UST (n = 21)	VDZ to ADA (n = 64)	VDZ to IFX (n = 119)	VDZ to UST (n = 30)
Demographic										
Age, y	43.7 (16.5)	41.8 (15.8)	43.7 (15.6)	40.1 (16.2)	41.8 (15.2)	45.2 (18.2)	40.2 (14.1)	47.1 (16.1)	47.2 (18.5)	47.3 (16.1)
Sex										
Female	50.5	49.1	47.9	60.6	53.0	53.2	61.9	45.3	46.2	50.0
Male	49.5	50.9	52.1	39.4	47.0	46.8	38.1	54.7	53.8	50.0
Race/ethnicity										
Asian	1.8	1.5	1.5	3.0	1.5	2.1	0.0	3.1	2.5	0.0
Black or African American	5.2	6.4	3.0	0.0	8.3	5.1	4.8	3.1	5.0	6.7
White or Caucasian	88.1	88.8	91.5	90.9	85.0	86.1	85.7	85.9	89.1	86.7
Unknown or other	4.9	3.3	4.0	6.1	5.3	6.7	9.5	7.8	3.4	6.7
Insurance type										
Commercial	65.9	66.1	69.8	75.8	66.2	65.2	61.9	56.3	53.8	76.7
Medicaid	6.8	8.5	4.5	3.0	7.1	9.4	4.8	3.1	6.7	0.0
Medicare	7.9	7.9	5.5	0.0	5.3	10.4	4.8	9.4	16.0	6.7
Commercial/Medicaid	2.7	3.0	2.0	0.0	3.4	2.1	14.3	4.7	2.5	0.0
Commercial/Medicare	3.8	2.7	4.5	3.0	2.6	4.3	4.8	3.1	6.7	3.3
Medicaid/Medicare	0.7	0.3	1.0	0.0	0.8	0.8	0.0	0.0	1.7	0.0
Commercial/Medicaid/ Medicare	0.1	0.0	0.0	0.0	0.4	0.3	0.0	0.0	0.0	0.0
Uninsured	1.4	0.9	0.5	9.1	3.8	0.5	0.0	1.6	0.8	3.3
Missing, unknown, or other	10.6	10.6	12.2	9.1	10.5	7.0	9.5	21.9	11.8	10.0
Clinical characteristic										
Conditions in the pre–index period^b^										
Cardiovascular disease	25.9	22.1	22.4	24.2	28.2	31.8	23.8	21.9	27.7	23.3
Mental disorder	19.2	16.1	19.2	33.3	24.1	20.9	9.5	18.8	14.3	3.3
Chronic pulmonary disease	12.5	10.9	12.0	6.1	15.4	13.1	9.5	12.5	15.1	0.0
Diabetes mellitus	7.3	7.3	6.5	3.0	5.6	9.1	9.5	4.7	10.1	10.0
Liver disease	6.4	8.2	5.2	3.0	6.4	7.0	0.0	4.7	6.7	6.7
CCI score	0.5 (1.0)	0.4 (0.9)	0.5 (1.0)	0.2 (0.5)	0.5 (1.0)	0.5 (1.0)	0.7 (1.0)	0.8 (1.2)	0.8 (1.3)	0.6 (1.1)
Body mass index, kg/m^2^	27.3 (6.3)	27.8 (6.5)	27.7 (6.4)	27.4 (6.2)	27.2 (6.8)	26.9 (6.3)	25.7 (5.1)	26.1 (5.3)	27.4 (5.6)	26.2 (5.9)
Smoking	16.0	18.5	12.5	18.2	17.7	18.5	14.3	10.9	14.3	6.7
Disease extent										
Pancolitis	45.2	47.3	40.9	45.5	44.7	47.1	47.6	45.3	53.8	26.7
Left-sided disease	9.8	8.8	12.5	0.0	11.7	9.1	4.8	12.5	5.9	3.3
Proctosigmoiditis	7.9	8.5	12.0	9.1	2.6	5.6	4.8	9.4	8.4	20.0
Proctitis, other, and unspecified	37.0	35.5	34.7	45.5	41.0	38.2	42.9	32.8	31.9	50.0
Duration of conventional therapy										
0 d	13.0	8.8	11.7	21.2	10.5	15.2	23.8	7.8	20.2	36.7
1-30 d	12.1	11.5	9.5	12.1	12.4	16.3	4.8	3.1	14.3	13.3
31-60 d	12.0	11.2	12.7	9.1	13.5	11.8	19.1	15.6	8.4	3.3
61-90 d	9.9	11.2	9.2	9.1	7.9	11.8	4.8	7.8	9.2	6.7
≥91 d	53.1	57.3	56.9	48.5	55.6	44.9	47.6	65.6	47.9	40.0

Values are mean (SD) or %.

Abbreviations: ADA, adalimumab; CCI, Charlson Comorbidity Index; IFX, infliximab; UST, ustekinumab; VDZ, vedolizumab.

^a^Including 2 patients with ulcerative colitis who received UST as a first-line biologic.

^b^The 5 most prevalent conditions in the total ulcerative colitis cohort.

### Treatment Duration

In patients with CD, the mean length of follow-up was 1488.8 (SD = 652.6) days. Treatment duration, defined as total treatment time including restarting after discontinuation, is shown in Table 4. The median duration of the first line of biologic treatment for those who received ustekinumab (106.5 days) was shorter than those who received adalimumab (266.0 days), infliximab (272.0 days), or vedolizumab (269.0 days). Similarly, in patients with CD, the median duration of the second line of biologic treatment for those who received ustekinumab (235.0 days) was shorter than for those who received adalimumab (318.0 days), infliximab (313.5 days), or vedolizumab (318.0 days).

**Table 4.** Duration of each line of treatment.

	Treatment duration (d)			
	Adalimumab	Infliximab	Vedolizumab	Ustekinumab
Crohn’s disease				
First line	1605	956	329	118
Mean (SD)	435.2 (457.1)	411.6 (408.9)	413.1 (375.8)	206.6 (293.0)
Median	266.0	272.0	269.0	106.5
Second line	553	720	886	849
Mean (SD)	454.2 (455.1)	459.7 (447.3)	458.1 (425.3)	353.6 (327.3)
Median	318.0	313.5	318.0	235.0
Ulcerative colitis				
First line	764	661	213	NA
Mean (SD)	279.0 (328.7)	325.1 (352.8)	258.3 (293.8)	NA
Median	167.0	213.0	161.0	NA
Second line	330	450	776	84
Mean (SD)	419.6 (474.1)	423.6 (445.8)	459.5 (448.7)	240.1 (311.7)
Median	237.5	239.5	288.0	98.5

Values are n, unless otherwise indicated.

Abbreviations: NA, not applicable.

In patients with UC, the mean length of follow-up was 1309.2 (SD = 642.9) days. The median duration of the first line of biologic treatment for those who received infliximab (213.0 days) was longer than for those who received adalimumab (167.0 days) or vedolizumab (161.0 days). In patients with UC, the median duration of the second line of biologic treatment was similar for those who received adalimumab (237.5 days) and infliximab (239.5 days) but was longer for those who received vedolizumab (288.0 days) and shorter for those who received ustekinumab (98.5 days). The mean length of follow-up was 1309.2 (SD = 642.9) days.

### Time to Switching, Discontinuation, or the End of the Study Period

**Figure 2.**
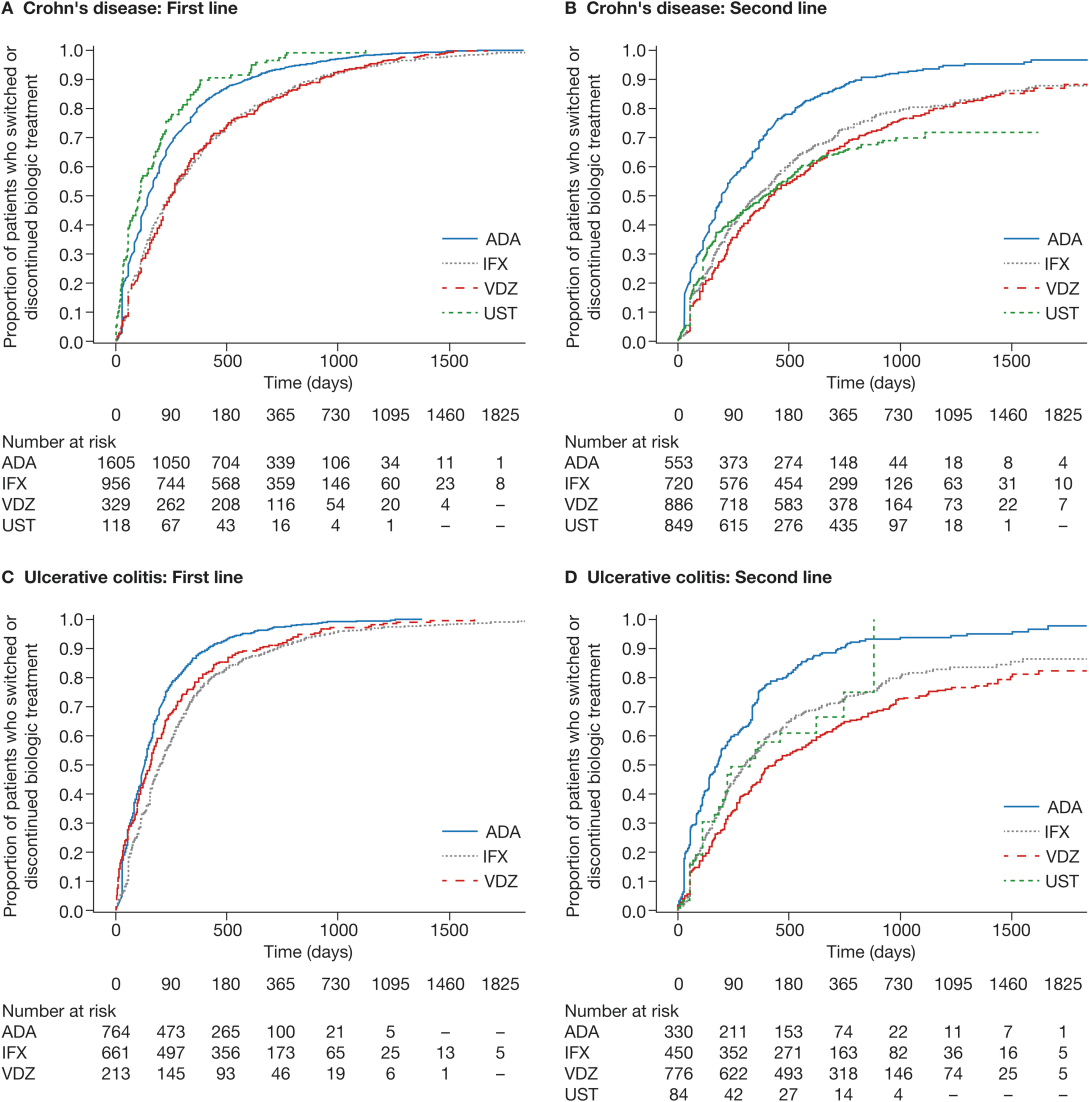
Time to switching, discontinuation, or the end of study period in (A) Crohn's disease: first line, (B) Crohn's disease: second line, (C) ulcerative colitis: first line, and (D) ulcerative colitis: second line. ADA, adalimumab; CI, confidence interval; IFX, infliximab; UST, ustekinumab; VDZ, vedolizumab.

After 365 days of initiating a first-line biologic, 79.9%, 62.6%, 65.1%, and 86.4% of patients with CD receiving adalimumab, infliximab, vedolizumab, or ustekinumab, respectively, as first-line biologics, had discontinued or switched treatment (Figure 2A). After 365 days of initiating a second-line biologic, the proportion of patients who had switched or discontinued a second-line biologic was lower for vedolizumab (45.2%) than for adalimumab (68.4%), infliximab (50.3%), and ustekinumab (48.3%; Figure 2B).

In patients with UC, by day 365 of initiating a first-line biologic, 87.6%, 74.3%, and 78.4% of patients receiving adalimumab, infliximab, or vedolizumab, respectively, as first-line biologics, had discontinued or switched (Figure 2C). After 365 days of initiating a second-line biologic, the proportion of patients who had switched or discontinued a second-line biologic was lower for vedolizumab (44.4%) than for adalimumab (74.6%), infliximab (54.7%), and ustekinumab (57.9%) (Figure 2D).

### Adjusted Rate of Switching or Discontinuation

**Figure 3.**
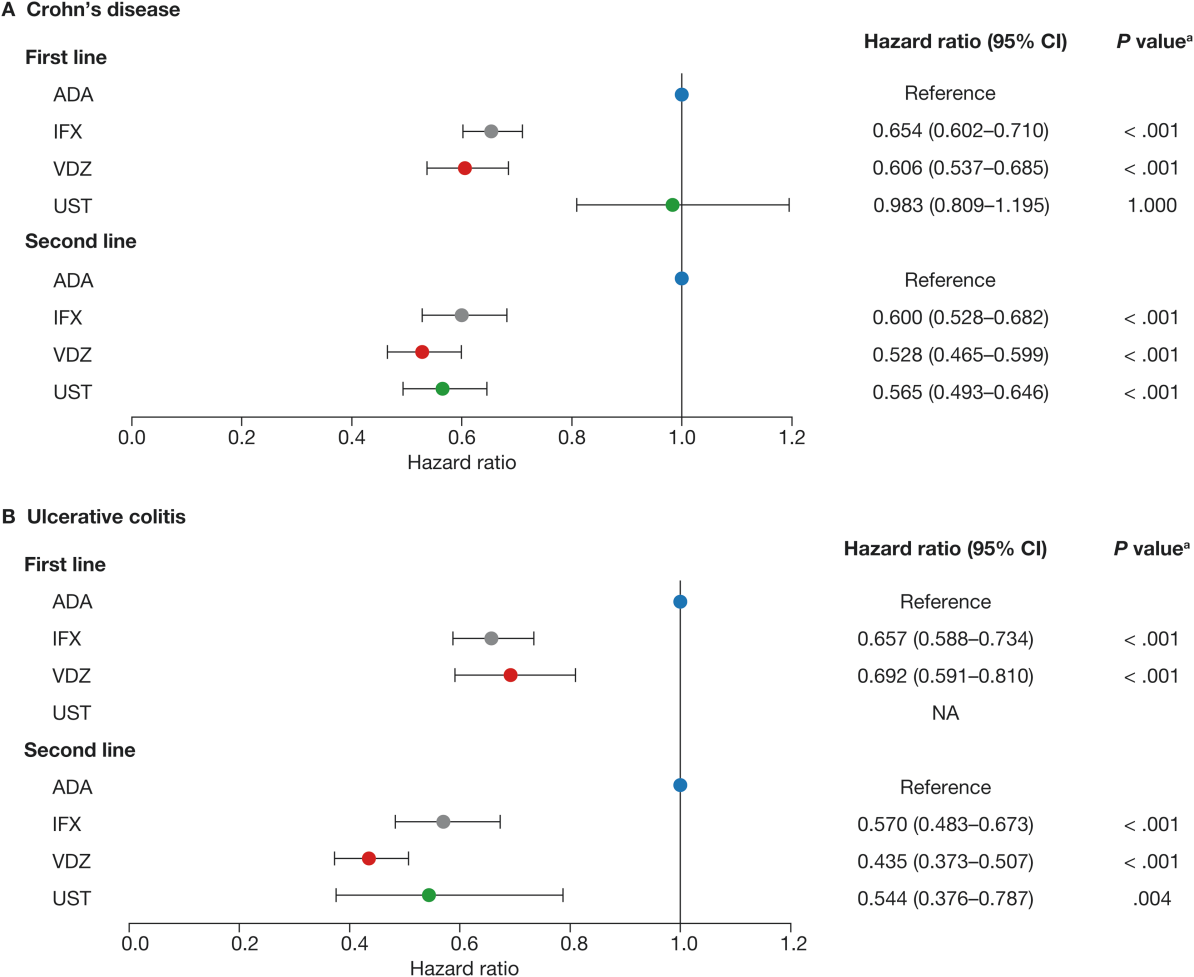
Adjusted rate of switching or discontinuation of first- and second-line biologics. ^a^Bonferroni adjusted. Abbreviations: ADA, adalimumab; CI, confidence interval; IFX, infliximab; NA, not applicable; UST, ustekinumab; VDZ, vedolizumab.

After adjustment for baseline demographics and clinical characteristics, as first-line biologics, vedolizumab and infliximab had a 39.4% (HR, 0.606; 95% confidence interval [CI], 0.537-0.685; *P < .*001) and 34.6% (HR, 0.654; 95% CI, 0.602-0.710; *P < .*001) lower rate of switching or discontinuation, respectively, than adalimumab in patients with CD (Figure 3A). The rate of switching or discontinuation for ustekinumab as a first-line biologic was not significantly different from adalimumab (HR, 0.983; 95% CI, 0.809-1.195; *P = *1.000). As second-line biologics, vedolizumab, ustekinumab, and infliximab had a 47.2% (HR, 0.528; 95% CI, 0.465-0.599; *P < .*001), 43.5% (HR 0.565, 95% CI 0.493-0.646; *P < .*001), and 40.0% (HR, 0.600; 95% CI, 0.528-0.682; *P < .*001) lower rate of switching or discontinuation, respectively, than adalimumab in patients with CD (Figure 3A; Supplementary Tables 3 and 4). Patients with CD who had 1 or 2 baseline all-cause hospitalizations had a significantly higher rate of switching or discontinuation for first- or second-line biologics compared with those who had no baseline hospitalizations (first line: HR, 1.127; 95% CI, 1.035-1.227; *P = *.006; second line: HR, 1.132; 95% CI, 1.022-1.254; *P = *.018). Patients who had ≥3 hospitalizations at baseline only had a significantly higher rate of switching or discontinuation for first- but not second-line biologics, compared with those who had no baseline hospitalizations (first line: HR, 1.193; 95% CI, 1.017-1.400; *P = *.031; second line: HR, 0.982; 95% CI, 0.806-1.197; *P = *.858). For rate of switching or discontinuation at the first line of biologic treatment, all index years were associated with a significantly higher rate than 2013 (*P < *.001). While at second line, baseline CCI score and the presence of perianal disease were associated with significantly lower and higher rates of discontinuation or switching, respectively (CCI score: HR, 0.930; 95% CI, 0.888-0.973; *P = *.02; perianal disease: HR, 1.251; 95% CI, 1.002-1.561; *P = *.048).

Adjusted analysis of each biologic led to the identification of variables that significantly impacted the rate of switching or discontinuation of some first- or second-line biologic treatments for patients with CD (Supplementary Tables 5 and 6). Notably, for patients receiving adalimumab as a first-line biologic, the presence of 1 or more baseline all-cause hospitalizations, compared with none, led to a significantly higher rate of discontinuation or switching. Patients of a race or ethnicity other than White or Caucasian who were receiving adalimumab as a first-line biologic also had a significantly higher rate of discontinuation or switching compared with patients who were White or Caucasian. For patients receiving ustekinumab as a first-line biologic, male patients had a significantly lower rate of switching or discontinuation than female patients, while those who smoked at baseline had a significantly higher rate. At the second line of biologic treatment, increasing baseline CCI score resulted in a significantly lower rate of switching or discontinuation for patients receiving infliximab or vedolizumab. For patients receiving ustekinumab at the second line of biologic treatment, the presence of perianal disease was associated with a significantly higher rate of switching or discontinuation, while fistulas were associated with a lower rate. Receipt of conventional therapy for 91 days or more was associated with a higher rate of switching or discontinuation for patients receiving infliximab as a second-line biologic, and the presence of mental disorders at baseline was associated with a higher rate of switching or discontinuation for patients receiving ustekinumab as a second-line biologic. Finally, patients of a race or ethnicity other than White or Caucasian who were receiving infliximab as a second-line biologic had a lower rate of switching or discontinuation compared with patients who were White or Caucasian.

As first-line biologics, infliximab and vedolizumab had a 34.3% (HR, 0.657; 95% CI, 0.588-0.734; *P < .*001) and 30.8% (HR, 0.692; 95% CI, 0.591-0.810; *P < .*001) lower rate of switching or discontinuation, respectively, than adalimumab in patients with UC (Figure 3B). As second-line biologics, vedolizumab, ustekinumab, and infliximab had a 56.5% (HR, 0.435; 95% CI, 0.373-0.507; *P < .*001), 45.6% (HR, 0.544; 95% CI, 0.376-0.787; *P = .*004), and 43.0% (HR, 0.570; 95% CI, 0.483-0.673; *P < .*001) lower rate of switching or discontinuation, respectively, than adalimumab in patients with UC (Figure 3B; Supplementary Tables 7 and 8).

At the first line of biologic treatment, for patients with UC, all index years were associated with a significantly higher rate of switching or discontinuation than 2013 (*P < *.001 for all years except 2015 [*P = *.02]). At second line, only the years 2016 and 2017 were associated with a significantly higher rate of switching or discontinuation compared with 2013 (2016: HR, 1.307; 95% CI, 1.034-1.653; *P = *.025; 2017: HR, 1.435; 95% CI, 1.118-1.841; *P = *.005). Patients who were Black or African American, Asian, or of other race/ethnicity had a significantly lower rate of switching or discontinuation at second line compared with White or Caucasian patients (HR, 0.740; 95% CI, 0.606-0.904; *P = *.003).

Adjusted analysis of each biologic demonstrated some variables that significantly impacted the rate of switching or discontinuation for some first- and second-line biologic treatments for patients with UC (Supplementary Tables 9 and 10). Baseline disease extent other than pancolitis was associated with a significantly higher rate of switching or discontinuation for patients receiving infliximab as a first-line biologic. At the second line of biologic treatment, patients of a race or ethnicity other than White or Caucasian who were receiving adalimumab had a significantly lower rate of switching or discontinuation. Increasing age and duration of conventional therapy at baseline of 91 days or above resulted in a significantly lower rate of switching or discontinuation for patients who were receiving infliximab, while disease extent other than pancolitis significantly increased the rate of switching or discontinuation for patients receiving vedolizumab.

## Discussion

Randomized controlled trials are considered the gold standard for evaluating the efficacy of biologics for IBD treatment; however, data from clinical trials provide only limited insights into the impact of treatment sequence on outcomes.^14^ Real-world data provide an opportunity to identify patients with IBD who have received multiple lines of biologics, but these analyses are limited by a lack of data on clinical remission and response.^14^ This study was devised to gain insight into biologic treatment sequences and their success, as demonstrated by persistence on treatment, in patients with IBD who received ≥2 biologics successively.

Most patients with CD or UC received an anti-TNFα treatment followed by vedolizumab or another anti-TNFα treatment, which is consistent with results of other work investigating biologic treatment sequences in clinical practice.^15,16^ The majority of patients with CD received adalimumab as a first-line biologic, while vedolizumab and ustekinumab were common second-line biologics. For patients with UC, both adalimumab and infliximab were common first-line biologics. Only 13% of patients received vedolizumab as a first-line biologic in patients with UC, although vedolizumab was the most common second-line biologic. The American Gastrointestinal Association clinical guidelines suggest using infliximab or vedolizumab over adalimumab for the induction and maintenance of remission in biologic-naive patients with moderately to severely active UC, although adalimumab is considered a reasonable alternative.^12^ In addition to clinical guidelines, payer access may have influenced access to certain biologics. Use of ustekinumab was low among patients with UC, and it was almost exclusively used as a second-line biologic. This was not unexpected, as the U.S. Food and Drug Administration approval for use of ustekinumab in moderately to severely active UC was only obtained in 2019,^17^ and current clinical guidelines do not provide any recommendations regarding its use in these patients.^11,12^

Patients receiving vedolizumab or infliximab persisted on treatment for longer than those who received adalimumab as first- or second-line biologics for both CD and UC. In the VARSITY trial, vedolizumab demonstrated superior efficacy to adalimumab for clinical remission in patients with moderately to severely active UC who were biologic-naive or who were anti-TNFα treatment-experienced.^9^ Discontinuation was higher among patients in the adalimumab group than the vedolizumab group, with lack of efficacy and adverse events reported as common reasons for discontinuation.^9^ Assuming that our findings translate to a real-world setting, patients with UC may persist longer on vedolizumab than adalimumab owing to a greater clinical benefit, as demonstrated in the VARSITY trial.^9^

Ustekinumab had similar persistence to adalimumab as a first-line biologic in patients with CD but better persistence than adalimumab as a second-line biologic for both CD and UC. It is not apparent from this real-world study which factors drive similar persistence estimates between ustekinumab and adalimumab as first-line biologics in patients with CD, although it is reasonable to view these findings in context of the SEAVUE trial. In this trial, ustekinumab and adalimumab achieved similar rates of clinical remission in biologic-naive patients with moderately to severely active CD.^18^ Ustekinumab and adalimumab may, therefore, have similar persistence as first-line biologics in a real-world setting owing to similar clinical benefit.

This study may allow decision makers to better understand the comparative effectiveness of biologics across treatment sequences in a real-world setting. The results suggest that patients with IBD could benefit from receiving infliximab or vedolizumab over adalimumab as first- or second-line biologics, and from receiving ustekinumab over adalimumab as a second-line biologic. In line with some of these considerations, a review of treatment sequencing of biologics for IBD suggested that patients with CD or UC should be treated with a non–anti-TNFα treatment, such as vedolizumab, prior to exposure to anti-TNFα treatment. The study also concluded that, after anti-TNFα treatment failure, switching from one anti-TNFα treatment to another should be avoided. Regarding ustekinumab, the review, based on data from clinical trials, suggested that treatment may be given to either patients with CD or UC who are anti-TNFα treatment-naive or treatment-experienced.^19^

The study population included patients with varied baseline demographics and clinical characteristics, which were used as confounders for adjustment of the estimates. With these confounders accounted for, choice of biologic was a significant driver of persistence. The Optum Clinical Database patient population is representative of the general U.S. population with regard to age, sex, and race/ethnicity; therefore, the results of this study should be broadly generalizable.

As the ROTARY study was a retrospective, observational cohort study conducted using EHR data, the following limitations should be considered when interpreting the results. In addition to use of onsite administration records, medication use was imputed from prescription orders, which may be incomplete or contain errors. While EHR data may not capture all prescription orders, medication administrations, and procedure records from providers outside of the EHR system, we expect that this had a limited impact given the longitudinal nature the study, which required multiple records to define treatment sequences over time. This study did not examine laboratory test results, which could be confounders, but it was assumed that other clinical variables, including prior hospitalization, disease extent or location, and disease characteristics, would compensate for this absence. Lastly, the reason for treatment switching was not captured by the database, thereby restricting the scope of this analysis. Future studies are required to investigate the reasons for variability in persistence between biologic treatments.

## Conclusions

Adalimumab was the most common first-line biologic among patients with IBD who received 2 biologics successively in clinical practice; however, there were notable differences in persistence between biologic treatments, favoring vedolizumab, infliximab, and ustekinumab over adalimumab. These data supplement existing evidence on the comparative effectiveness of biologics and may help to inform treatment choice and sequencing of biologic treatments in IBD.

## Supplementary data

Supplementary data is available at *Inflammatory Bowel Diseases* online.

izad245_suppl_Supplementary_Material
