# Supplementary material for: Real-World Persistence of Successive Biologics in Patients With Inflammatory Bowel Disease: Findings From ROTARY
Source: Inflamm Bowel Dis. 2023 Oct 31;30(10):1776–87. doi: 10.1093/ibd/izad245 (PMC11447059; doi:10.1093/ibd/izad245)
Supplement: izad245_suppl_Supplementary_Material [file izad245_suppl_supplementary_material.docx]

# Supplementary Material

## Supplementary Table 1. Patient attrition for the Crohn’s disease cohort.

| Criterion | | Remaining | | Excluded | |
| --- | --- | --- | --- | --- | --- |
|  |  | **n** | **%** | **n** | **%** |
| Evidence of treatment | ≥ 1 prescription or administration of a biologic^a^ during the patient identification period [January 1, 2013 to February 29, 2020] | 150 631 | – | – | – |
|  | Receiving only one biologic on initial therapy date | 150 506 | 99.9 | 125 | 0.1 |
| EHR activity | EHR activity before the start of the baseline period [365 days before the index date] | 127 931 | 85.0 | 22 575 | 15.0 |
| Crohn’s disease diagnosis | ≥ 2 diagnoses of Crohn’s disease during the baseline period^b^ | 19 960 | 15.6 | 107 971 | 84.4 |
|  | ≥ 1 diagnosis of Crohn’s disease during the follow-up period | 19 447 | 97.4 | 513 | 2.6 |
| New start | Exclusion: ≥ 1 prescription or administration of a biologic^a^ during the baseline period | 16 421 | 84.4 | 3026 | 15.6 |
| No other conditions | Exclusion: ≥ 1 diagnosis for rheumatoid arthritis, psoriatic arthritis, ankylosing spondylitis, plaque psoriasis, hidradenitis suppurativa, or noninfectious uveitis in the 6 months before the index date | 15 743 | 95.9 | 678 | 4.1 |
| Adult | ≥ 18 years old at index date with valid demographic information | 13 661 | 86.8 | 2082 | 13.2 |
| ≥ 2 qualifying lines of therapy | ≥ 1 prescription or administration of second biologic^a^ following the first biologic | 3028 | 22.2 | 10 633 | 77.8 |
|  | Monotherapy during second line of treatment | 3008 | 99.3 | 20 | 0.7 |

^a^The biologics included were adalimumab, ustekinumab, infliximab, or vedolizumab.

^b^For patients with diagnoses of both Crohn’s disease and ulcerative colitis, ≥ 3 consecutive Crohn’s disease diagnoses after an ulcerative colitis diagnosis and ≥ 3 ulcerative colitis diagnoses after a Crohn’s disease diagnosis were used to classify patients to the Crohn’s disease and ulcerative colitis cohorts, respectively.

Abbreviation: EHR, electronic health record.

## Supplementary Table 2. Patient attrition for the ulcerative colitis cohort.

| Criterion | | Remaining | | Excluded | |
| --- | --- | --- | --- | --- | --- |
|  |  | **n** | **%** | **n** | **%** |
| Evidence of treatment | ≥ 1 prescription or administration of a biologic^a^ during the patient identification period [January 1, 2013 to February 29, 2020] | 150 631 | – | – | – |
|  | Receiving only one biologic on initial therapy date | 150 506 | 99.9 | 125 | 0.1 |
| EHR activity | EHR activity before the start of the baseline period [365 days before the index date] | 127 931 | 85.0 | 22 575 | 15.0 |
| Ulcerative colitis diagnosis | ≥ 2 diagnoses of ulcerative colitis during the baseline period^b^ | 9451 | 7.4 | 118 480 | 92.6 |
|  | ≥ 1 diagnosis of ulcerative colitis during the follow-up period | 9049 | 95.7 | 402 | 4.3 |
| New start | Exclusion: ≥ 1 prescription or administration of a biologic^a^ during the baseline period | 8186 | 90.5 | 863 | 9.5 |
| No other conditions | Exclusion: ≥ 1 diagnosis for rheumatoid arthritis, psoriatic arthritis, ankylosing spondylitis, plaque psoriasis, hidradenitis suppurativa, or noninfectious uveitis in the 6 months before the index date | 7893 | 96.4 | 293 | 3.6 |
| Adult | ≥ 18 years old at index date with valid demographic information | 7123 | 90.2 | 770 | 9.8 |
| ≥ 2 qualifying lines of therapy | ≥ 1 prescription or administration of second biologic^a^ following the first biologic | 1654 | 23.2 | 5469 | 76.8 |
|  | Monotherapy during second line of treatment | 1640 | 99.2 | 14 | 0.8 |

^a^The biologics included were adalimumab, ustekinumab, infliximab, or vedolizumab.

^b^For patients with diagnoses of both Crohn’s disease and ulcerative colitis, ≥ 3 consecutive Crohn’s disease diagnoses after an ulcerative colitis diagnosis and ≥ 3 ulcerative colitis diagnoses after a Crohn’s disease diagnosis were used to classify patients to the Crohn’s disease and ulcerative colitis cohorts, respectively.

Abbreviation: EHR, electronic health record.

## Supplementary Table 3. Cox proportional hazard model: Rate of switching or discontinuation of a first-line biologic in patients with Crohn’s disease.

| Independent variable | Hazard ratio (95% CI) | *P* value | Bonferroni-adjusted *P* value |
| --- | --- | --- | --- |
| First-line biologic |  | < .001 | – |
| Adalimumab | Reference | – | – |
| Infliximab | 0.654 (0.602–0.710) | < .001 | < .001 |
| Vedolizumab | 0.606 (0.537–0.685) | < .001 | < .001 |
| Ustekinumab | 0.983 (0.809–1.195) | .864 | 1.000 |
| Age | 0.998 (0.995–1.000) | .081 |  |
| Sex |  |  |  |
| Female | Reference | – | – |
| Male | 0.936 (0.869–1.008) | .081 | – |
| Race/ethnicity |  |  |  |
| White or Caucasian | Reference | – | – |
| Black or African American, Asian, unknown/other | 1.052 (0.937–1.182) | .390 | – |
| Body mass index, kg/m^2^ |  |  | – |
| Missing, < 18.5–24.9 | Reference | – | – |
| ≥ 25 | 0.980 (0.910–1.056) | .604 | – |
| Baseline smoking | 1.087 (0.988–1.197) | .087 |  |
| Baseline Crohn’s disease-related conditions^a^ |  |  | – |
| Perianal disease | 0.965 (0.800–1.164) | .710 | – |
| Fistula | 1.002 (0.885–1.134) | .976 | – |
| Baseline disease location |  |  |  |
| Ileum-colon | Reference | – | – |
| Other | 1.041 (0.963–1.125) | .309 | – |
| Baseline extraintestinal manifestations | 1.005 (0.869–1.161) | .951 | – |
| Baseline all-cause hospitalization |  |  |  |
| 0 | Reference | – | – |
| 1–2 | 1.127 (1.035–1.227) | .006 | – |
| ≥ 3 | 1.193 (1.017–1.400) | .031 | – |
| Baseline CCI score | 1.007 (0.974–1.042) | .678 | – |
| Baseline mental disorder | 1.080 (0.988–1.179) | .089 | – |
| Baseline conventional therapy duration, days |  |  |  |
| 0–90 | Reference | – | – |
| ≥ 91 | 1.021 (0.946–1.101) | .598 | – |
| Index year |  |  |  |
| 2013 | Reference | – | – |
| 2014 | 1.317 (1.157–1.499) | < .001 | – |
| 2015 | 1.244 (1.094–1.414) | < .001 | – |
| 2016 | 1.444 (1.271–1.641) | < .001 | – |
| 2017 | 1.694 (1.476–1.945) | < .001 | – |
| 2018 | 2.223 (1.913–2.583) | < .001 | – |
| 2019/2020 | 3.414 (2.858–4.079) | < .001 | – |

Analyses were adjusted for each variable listed in the table. N = 3008; proportional hazards test (overall−log[time] with Schoenfeld residuals): *P* value < .001.

^a^Stricture not included owing to low rate; abscess not included owing to correlation with perianal disease.

Abbreviations: CCI, Charlson Comorbidity Index.

## Supplementary Table 4. Cox proportional hazard model: Rate of switching or discontinuation of a second-line biologic in patients with Crohn’s disease.

| Independent variable | Hazard ratio (95% CI) | *P* value | Bonferroni-adjusted *P* value |
| --- | --- | --- | --- |
| Second-line biologic^a^ |  | < .001 | – |
| Adalimumab | Reference | – | – |
| Infliximab | 0.600 (0.528–0.682) | < .001 | < .001 |
| Vedolizumab | 0.528 (0.465–0.599) | < .001 | < .001 |
| Ustekinumab | 0.565 (0.493–0.646) | < .001 | < .001 |
| Age | 1.001 (0.998–1.004) | .417 | – |
| Sex | | | |
| Female | Reference | – | – |
| Male | 0.994 (0.907–1.090) | .901 | – |
| Race/ethnicity | | | |
| White or Caucasian | Reference | – | – |
| Black or African American, Asian, unknown/other | 0.920 (0.796–1.063) | .256 | – |
| Body mass index, kg/m^2^ | | | |
| Missing, < 18.5–24.9 | Reference | – | – |
| ≥ 25 | 0.965 (0.881–1.057) | .448 | – |
| Baseline smoking | 1.016 (0.904–1.141) | .790 | – |
| Baseline Crohn’s disease-related conditions^b^ | | | |
| Perianal disease | 1.251 (1.002–1.561) | .048 | – |
| Fistula | 0.980 (0.840–1.142) | .793 | – |
| Baseline disease location | | | |
| Ileum-colon | Reference | – | – |
| Other | 1.078 (0.978–1.188) | .130 | – |
| Baseline extraintestinal manifestations | 1.018 (0.849–1.220) | .851 | – |
| Baseline all-cause hospitalization | | | |
| 0 | Reference | – | – |
| 1–2 | 1.132 (1.022–1.254) | .018 | – |
| ≥ 3 | 0.982 (0.806–1.197) | .858 | – |
| Baseline CCI score | 0.930 (0.888–0.973) | .002 | – |
| Baseline mental disorder | 1.022 (0.916–1.139) | .698 | – |
| Baseline conventional therapy duration, days | | | |
| 0–90 | Reference | – | – |
| ≥ 91 | 1.082 (0.986–1.187) | .095 | – |
| Index year | | | |
| 2013 | Reference | – | – |
| 2014 | 1.062 (0.919–1.228) | .413 | – |
| 2015 | 1.066 (0.922–1.232) | .387 | – |
| 2016 | 1.041 (0.898–1.208) | .594 | – |
| 2017 | 0.980 (0.828–1.158) | .809 | – |
| 2018 | 0.886 (0.723–1.087) | .246 | – |
| 2019/2020 | 0.699 (0.496–0.986) | .041 | – |

Analyses were adjusted for each variable listed in the table. N = 3008; proportional hazards test (overall−log[time] with Schoenfeld residuals): *P* value = .002.

^a^First-line biologics are adalimumab, infliximab, vedolizumab, and ustekinumab.

^b^Stricture not included owing to low rate; abscess not included owing to correlation with perianal disease.

Abbreviations: CCI, Charlson Comorbidity Index.

## Supplementary Table 5. Cox proportional hazard model: Rate of switching or discontinuation of each individual first-line biologic in patients with Crohn’s disease.

| **Independent variable** | **First-line biologic treatment** | | | | | | | |
| --- | --- | --- | --- | --- | --- | --- | --- | --- |
|  | **Adalimumab** | | **Infliximab** | | **Ustekinumab** | | **Vedolizumab** | |
|  | **Hazard ratio  (95% CI)** | ***P* value** | **Hazard ratio  (95% CI)** | ***P* value** | **Hazard ratio  (95% CI)** | ***P* value** | **Hazard ratio  (95% CI)** | ***P* value** |
| Age | 0.998  (0.994–1.001) | .2088127548 | 0.997  (0.993–1.002) | .2284137890 | 0.997  (0.981–1.012) | .6741775266 | 0.995  (0.986–1.003) | .1978334753 |
| Sex |  |  |  |  |  |  |  |  |
| Female | Reference | – | Reference | – | Reference | – | Reference | – |
| Male | 0.973  (0.879–1.078) | .6037949699 | 0.881  (0.771–1.007) | .0624666378 | 0.580  (0.368–0.915) | .0192929084 | 0.931  (0.732–1.184) | .5603885464 |
| Race/ethnicity |  |  |  |  |  |  |  |  |
| White or Caucasian | Reference | – | Reference | – | Reference | – | Reference | – |
| Black or African American, Asian, unknown/other | 1.229  (1.036–1.457) | .0177651914 | 0.988  (0.814–1.198) | .8989117345 | 0.711  (0.398–1.269) | .2483287498 | 0.962  (0.665–1.391) | .8363546524 |
| Body mass index, kg/m^2^ |  |  |  |  |  |  |  |  |
| Missing, < 18.5–24.9 | Reference | – | Reference | – | Reference | – | Reference | – |
| ≥ 25 | 0.995  (0.897–1.103) | .9196199337 | 1.009  (0.883–1.153) | .8952474584 | 0.883 (0.561–1.391) | .5925493397 | 0.854  (0.681–1.069) | .1681368688 |
| Baseline smoking | 1.108  (0.971–1.263) | .1274847622 | 1.002  (0.847–1.185) | .9848774803 | 3.511 (1.919–6.424) | .0000462466 | 1.064  (0.769–1.473) | .7082456100 |
| Baseline Crohn’s disease-related conditions^a^ |  |  |  |  |  |  |  |  |
| Perianal disease | 1.103  (0.815–1.493) | .5261587499 | 0.963  (0.729–1.273) | .7904270819 | 0.506  (0.182–1.409) | .1922677867 | 1.104  (0.613–1.991) | .7412442252 |
| Fistula | 1.036  (0.861–1.247) | .7055908301 | 1.021  (0.836–1.248) | .8357522384 | 1.092  (0.512–2.328) | .8198523602 | 0.795  (0.541–1.167) | .2420049571 |
| Baseline disease location |  |  |  |  |  |  |  |  |
| Ileum-colon | Reference | – | Reference | – | Reference | – | Reference | – |
| Other | 1.049  (0.943–1.167) | .3774239130 | 1.003  (0.871–1.155) | .9664403001 | 1.043  (0.671–1.619) | .8524192770 | 1.142  (0.902–1.446) | .2693526479 |
| Baseline extraintestinal manifestations | 0.986  (0.807–1.204) | .8886199400 | 0.985  (0.735–1.320) | .9205949899 | 1.711  (0.741–3.951) | .2085139563 | 1.160  (0.787–1.709) | .4544385592 |
| Baseline all-cause hospitalization |  |  |  |  |  |  |  |  |
| 0 | Reference | – | Reference | – | Reference | – | Reference | – |
| 1–2 | 1.168  (1.039–1.313) | .0092712966 | 1.142  (0.982–1.329) | .0843003315 | 0.760  (0.416–1.388) | .3711983790 | 1.106  (0.839–1.458) | .4755654277 |
| ≥ 3 | 1.296  (1.017–1.651) | .0359901876 | 1.283  (0.984–1.674) | .0655337458 | 0.550  (0.201–1.507) | .2451736974 | 0.816  (0.502–1.326) | .4117869783 |
| Baseline CCI score | 0.982  (0.933–1.033) | .4874583470 | 1.018  (0.962–1.077) | .5465641799 | 1.100  (0.912–1.326) | .3190247095 | 1.065  (0.972–1.167) | .1769952780 |
| Baseline mental disorder | 1.081  (0.958–1.221) | .2066034346 | 1.097  (0.934–1.288) | .2581803772 | 0.745  (0.452–1.226) | .2463210398 | 1.123  (0.846–1.489) | .4226503344 |
| Baseline conventional therapy duration, days |  |  |  |  |  |  |  |  |
| 0–90 | Reference | – | Reference | – | Reference | – | Reference | – |
| ≥ 91 | 0.999 (0.902–1.106) | .9795335557 | 1.088 (0.947–1.249) | .2329163090 | 0.662 (0.410–1.070) | .0918872046 | 1.127 (0.874–1.454) | .3562831602 |
| Index year |  |  |  |  |  |  |  |  |
| 2013 | Reference | – | Reference | – | Reference | – | Reference | – |
| 2014 | 1.183  (1.000–1.399) | .0497225803 | 1.618  (1.296–2.019) | .0000208322 | 1.299  (0.238–7.081) | .7620568257 | 0.169  (0.102–0.281) | < .0000000001 |
| 2015 | 1.038  (0.878–1.227) | .6642149827 | 1.639  (1.314–2.044) | .0000117364 | 4.177  (0.767–22.737) | .0981897031 | 0.219  (0.140–0.343) | < .0000000001 |
| 2016 | 1.263  (1.066–1.495) | .0069511155 | 1.724  (1.389–2.139) | .0000007525 | 3.738  (1.037–13.468) | .0438009433 | 0.291  (0.191–0.443) | .0000000081 |
| 2017 | 1.404  (1.171–1.682) | .0002393193 | 2.332  (1.827–2.977) | < .0000000001 | 1.988  (0.561–7.050) | .2872163817 | 0.439  (0.280–0.688) | .0003347737 |
| 2018 | 1.651  (1.356–2.010) | .0000006044 | 3.701  (2.815–4.867) | < .0000000001 | 4.245  (1.209–14.902) | .0240573229 | 0.500  (0.302–0.828) | .0070904037 |
| 2019/2020 | 2.136  (1.622–2.813) | .0000000645 | 5.410  (3.942–7.424) | < .0000000001 | 6.683  (1.858–24.035) | .0036255808 | – | – |

Analyses were adjusted for each variable listed in the table.

^a^Stricture not included due to low rate, abscess not included due to correlation with perianal disease.

Abbreviations: CCI, Charlson Comorbidity Index; CI, confidence interval.

## Supplementary Table 6. Cox proportional hazard model: Rate of switching or discontinuation of each individual second-line biologic in patients with Crohn’s disease.

| **Independent variable** | **Second-line biologic treatment** | | | | | | | |
| --- | --- | --- | --- | --- | --- | --- | --- | --- |
|  | **Adalimumab** | | **Infliximab** | | **Ustekinumab** | | **Vedolizumab** | |
|  | **Hazard ratio  (95% CI)** | ***P* value** | **Hazard ratio  (95% CI)** | ***P* value** | **Hazard ratio  (95% CI)** | ***P* value** | **Hazard ratio  (95% CI)** | ***P* value** |
| Age | 1.000  (0.993–1.008) | .9062432569 | 1.003  (0.997–1.009) | .2798371853 | 1.002  (0.995–1.008) | .6254486324 | 1.001  (0.995–1.006) | .7468397165 |
| Sex |  |  |  |  |  |  |  |  |
| Female | Reference | – | Reference | – | Reference | – | Reference | – |
| Male | 0.964  (0.796–1.168) | .7095968650 | 0.995  (0.830–1.194) | .9571731253 | 1.002  (0.821–1.223) | .9848513235 | 1.008  (0.846–1.202) | .9261678564 |
| Race/ethnicity |  |  |  |  |  |  |  |  |
| White or Caucasian | Reference | – | Reference | – | Reference | – | Reference | – |
| Black or African American, Asian, unknown/other | 0.824  (0.618–1.100) | .1898959002 | 0.729  (0.545–0.974) | .0326932349 | 1.087  (0.788–1.498) | .6122767985 | 1.094  (0.831–1.440) | .5214667809 |
| Body mass index, kg/m^2^ |  |  |  |  |  |  |  |  |
| Missing, < 18.5–24.9 | Reference | – | Reference | – | Reference | – | Reference | – |
| ≥ 25 | 1.064  (0.873–1.295) | .5392029288 | 0.854  (0.711–1.026) | .0924475973 | 0.996  (0.819–1.210) | .9639953309 | 1.016  (0.855–1.207) | .8580402847 |
| Baseline smoking | 1.069  (0.845–1.352) | .5780645226 | 0.903  (0.719–1.133) | .3769975158 | 1.200  (0.923–1.560) | .1727265038 | 0.992  (0.790–1.246) | .9444603850 |
| Baseline Crohn’s disease-related conditions^a^ |  |  |  |  |  |  |  |  |
| Perianal disease | 0.930  (0.601–1.438) | .7425790678 | 1.283  (0.794–2.072) | .3083959028 | 1.638  (1.062–2.528) | .0256868675 | 1.182  (0.745–1.874) | .4776113076 |
| Fistula | 1.071  (0.801–1.432) | .6444255278 | 1.036  (0.759–1.415) | .8230274508 | 0.693  (0.490–0.979) | .0372279724 | 1.119  (0.829–1.510) | .4626013827 |
| Baseline disease location |  |  |  |  |  |  |  |  |
| Ileum/colon | Reference | – | Reference | – | Reference | – | Reference | – |
| Other | 0.933  (0.758–1.148) | .5127441378 | 1.140  (0.936–1.387) | .1925148332 | 1.161  (0.949–1.420) | .1470676506 | 1.060  (0.881–1.276) | .5363017789 |
| Baseline extraintestinal manifestations | 1.044  (0.690–1.580) | .8374299341 | 1.084  (0.749–1.570) | .6692609036 | 0.898  (0.596–1.352) | .6057914650 | 1.135  (0.831–1.550) | .4255737423 |
| Baseline all-cause hospitalization |  |  |  |  |  |  |  |  |
| 0 | Reference | – | Reference | – | Reference | – | Reference | – |
| 1–2 | 1.175  (0.950–1.453) | .1377204849 | 1.173  (0.961–1.431) | .1161631798 | 0.949  (0.754–1.194) | .6545684469 | 1.187  (0.976–1.443) | .0857962381 |
| ≥ 3 | 0.892  (0.592–1.343) | .5832250954 | 0.928  (0.598–1.439) | .7372541027 | 1.143  (0.757–1.726) | .5257746199 | 0.950  (0.663–1.363) | .7820811188 |
| Baseline CCI score | 0.930  (0.844–1.025) | .1455817829 | 0.880  (0.790–0.980) | .0199011030 | 0.967  (0.883–1.059) | .4679873497 | 0.912  (0.837–0.994) | .0362162319 |
| Baseline mental disorder | 0.934  (0.729–1.196) | .5888218327 | 0.907  (0.732–1.124) | .3736707297 | 1.336  (1.064–1.678) | .0126712671 | 0.952  (0.781–1.162) | .6293077253 |
| Baseline conventional therapy duration, days |  |  |  |  |  |  |  |  |
| 0–90 | Reference | – | Reference | – | Reference | – | Reference | – |
| ≥ 91 | 1.033  (0.845–1.262) | .7536688704 | 1.232  (1.026–1.480) | .0252465133 | 0.979  (0.800–1.198) | .8377928760 | 1.053  (0.884–1.253) | .5650229869 |
| Index year |  |  |  |  |  |  |  |  |
| 2013 | Reference | – | Reference | – | Reference | – | Reference | – |
| 2014 | 0.773  (0.577–1.036) | .0848613363 | 1.096  (0.824–1.456) | .5290134074 | 1.012  (0.681–1.504) | .9527655840 | 1.401  (1.087–1.807) | .0092556897 |
| 2015 | 0.828  (0.619–1.109) | .2051731885 | 1.235  (0.930–1.640) | .1451009385 | 0.942  (0.645–1.376) | .7571392719 | 1.274  (0.981–1.653) | .0688457167 |
| 2016 | 0.878  (0.650–1.186) | .3952382325 | 1.369  (1.018–1.840) | .0373904524 | 0.928  (0.644–1.336) | .6873825435 | 1.042  (0.786–1.381) | .7760339819 |
| 2017 | 0.957  (0.674–1.359) | .8050946018 | 1.084  (0.774–1.520) | .6375474014 | 0.717  (0.478–1.075) | .1073027724 | 1.173  (0.863–1.594) | .3093478938 |
| 2018 | 0.632  (0.413–0.967) | .0345947635 | 1.058  (0.699–1.601) | .7907959300 | 0.824  (0.531–1.280) | .3899207298 | 0.854  (0.558–1.308) | .4686719852 |
| 2019/2020 | 0.587  (0.333–1.035) | .0657510489 | 0.713  (0.309–1.647) | .4286695965 | 0.537  (0.272–1.060) | .0732430569 | 0.885  (0.384–2.037) | .7736206503 |

Analyses were adjusted for each variable listed in the table.

^a^Stricture not included due to low rate, abscess not included due to correlation with perianal disease.

Abbreviations: CCI, Charlson Comorbidity Index; CI, confidence interval.

## Supplementary Table 7. Cox proportional hazard model: Rate of switching or discontinuation of a first-line biologic in patients with ulcerative colitis.

| Independent variables | Hazard ratio (95% CI) | *P* value | Bonferroni-adjusted *P* value | |
| --- | --- | --- | --- | --- |
| First-line biologic |  | < .001 | – | |
| Adalimumab | Reference | – | – |  |
| Infliximab | 0.657 (0.588–0.734) | < .001 | < .001 |  |
| Vedolizumab | 0.692 (0.591–0.810) | < .001 | < .001 |  |
| Age | 1.002 (0.999–1.006) | .141 | – |  |
| Sex | | | |  |
| Female | Reference | – | – |  |
| Male | 1.027 (0.929–1.136) | .601 | – |  |
| Race/ethnicity | | | |  |
| White or Caucasian | Reference | – | – |  |
| Black or African American, Asian, unknown/other | 0.899 (0.772–1.047) | .172 | – |  |
| Body mass index, kg/m^2^ | | | |  |
| Missing, < 18.5–24.9 | Reference | – | – |  |
| ≥ 25 | 0.942 (0.847–1.047) | .268 | – |  |
| Baseline smoking | 1.071 (0.929–1.234) | .346 | – |  |
| Baseline disease extent | | | |  |
| Pancolitis | Reference | – | – |  |
| Other | 1.040 (0.936–1.156) | .462 | – |  |
| Baseline extraintestinal manifestations | 0.889 (0.736–1.074) | .224 | – |  |
| Baseline all-cause hospitalization | | | |  |
| 0 | Reference | – | – |  |
| 1–2 | 1.088 (0.973–1.216) | .139 | – |  |
| ≥ 3 | 1.103 (0.875–1.390) | .405 | – |  |
| Baseline CCI score | 1.030 (0.978–1.085) | .263 | – |  |
| Baseline mental disorder | 0.988 (0.866–1.128) | .863 | – |  |
| Baseline conventional therapy duration, days | | | |  |
| 0–90 | Reference | – | – |  |
| ≥ 91 | 1.076 (0.975–1.188) | .145 | – |  |
| Index year | | | |  |
| 2013 | Reference | – | – |  |
| 2014 | 1.547 (1.240–1.931) | < .001 | – |  |
| 2015 | 1.391 (1.127–1.719) | .002 | – |  |
| 2016 | 1.630 (1.322–2.011) | < .001 | – |  |
| 2017 | 1.703 (1.364–2.125) | < .001 | – |  |
| 2018 | 2.159 (1.713–2.720) | < .001 | – |  |
| 2019/2020 | 3.613 (2.830–4.613) | < .001 | – |  |

Analyses were adjusted for each variable listed in the table. N = 1638; proportional hazards test (overall−log[time] with Schoenfeld residuals): *P* value < .001.

Abbreviations: CCI, Charlson Comorbidity Index.

## Supplementary Table 8. Cox proportional hazard model: Rate of switching or discontinuation of a second-line biologic in patients with ulcerative colitis.

| Independent variables | Hazard ratio (95% CI) | *P* value | Bonferroni-adjusted *P* value |
| --- | --- | --- | --- |
| Second-line biologic^a^ |  | < .001 | – |
| Adalimumab | Reference | – | – |
| Infliximab | 0.570 (0.483–0.673) | < .001 | < .001 |
| Vedolizumab | 0.435 (0.373–0.507) | < .001 | < .001 |
| Ustekinumab | 0.544 (0.376–0.787) | .001 | .004 |
| Age | 0.997 (0.993–1.001) | .109 | – |
| Sex | | | |
| Female | Reference | – | – |
| Male | 0.965 (0.853–1.092) | .576 | – |
| Race/ethnicity | | | |
| White or Caucasian | Reference | – | – |
| Black or African American, Asian, unknown/other | 0.740 (0.606–0.904) | .003 | – |
| Body mass index, kg/m^2^ | | | |
| Missing, < 18.5–24.9 | Reference | – | – |
| ≥ 25 | 1.018 (0.894–1.160) | .785 | – |
| Baseline smoking | 0.898 (0.751–1.074) | .238 | – |
| Baseline disease extent | | | |
| Pancolitis | Reference | – | – |
| Other | 1.148 (1.008–1.307) | .038 | – |
| Baseline extraintestinal manifestations | 1.049 (0.836–1.316) | .680 | – |
| Baseline all-cause hospitalization | | | |
| 0 | Reference | – | – |
| 1–2 | 1.148 (1.001–1.317) | .048 | – |
| ≥ 3 | 1.197 (0.897–1.598) | .222 | – |
| Baseline CCI score | 1.052 (0.991–1.117) | .099 | – |
| Baseline mental disorder | 0.959 (0.813–1.130) | .615 | – |
| Baseline conventional therapy duration, days | | | |
| 0–90 | Reference | – | – |
| ≥ 91 | 0.915 (0.810–1.034) | .153 | – |
| Index year | | | |
| 2013 | Reference | – | – |
| 2014 | 1.216 (0.953–1.551) | .117 | – |
| 2015 | 1.165 (0.920–1.476) | .204 | – |
| 2016 | 1.307 (1.034–1.653) | .025 | – |
| 2017 | 1.435 (1.118–1.841) | .005 | – |
| 2018 | 1.160 (0.872–1.543) | .307 | – |
| 2019/2020 | 1.247 (0.865–1.797) | .237 | – |

Analyses were adjusted for each variable listed in the table. N = 1638; proportional hazards test (overall–log[time] with Schoenfeld residuals): *P* value = .566.

^a^First-line biologics are adalimumab, infliximab, and vedolizumab.

Abbreviations: CCI, Charlson Comorbidity Index.

## Supplementary Table 9. Cox proportional hazard model: Rate of switching or discontinuation of each individual first-line biologic in patients with ulcerative colitis.

| **Independent variable** | **First-line biologic treatment** | | | | | |
| --- | --- | --- | --- | --- | --- | --- |
|  | **Adalimumab** | | **Infliximab** | | **Vedolizumab** | |
|  | **Hazard ratio (95% CI)** | ***P* value** | **Hazard ratio (95% CI)** | ***P* value** | **Hazard ratio (95% CI)** | ***P* value** |
| Age | 1.003 (0.998–1.008) | .2025450391 | 1.002 (0.997–1.006) | .5501632236 | 1.003 (0.994–1.013) | .5072616810 |
| Sex |  |  |  |  |  |  |
| Female | Reference | – | Reference | – | Reference | – |
| Male | 1.110 (0.954–1.291) | .1770507284 | 1.032 (0.877–1.215) | .7016481024 | 0.981 (0.727–1.324) | .8999455696 |
| Race/ethnicity |  |  |  |  |  |  |
| White or Caucasian | Reference | – | Reference | – | Reference | – |
| Black or African American, Asian, unknown/other | 0.905 (0.706–1.162) | .4356259963 | 0.837 (0.670–1.046) | .1181302753 | 1.113 (0.708–1.747) | .6431971095 |
| Body mass index, kg/m^2^ |  |  |  |  |  |  |
| Missing, < 18.5 – 24.9 | Reference | – | Reference | – | Reference | – |
| ≥ 25+ | 0.887 (0.757–1.039) | .1368029987 | 1.022 (0.864–1.209) | .7977341730 | 0.949 (0.692–1.301) | .7444332743 |
| Baseline smoking | 1.106 (0.890–1.376) | .3630237345 | 1.040 (0.834–1.296) | .7278550779 | 0.924 (0.587–1.456) | .7345419294 |
| Baseline disease extent |  |  |  |  |  |  |
| Pancolitis | Reference | – | Reference | – | Reference | – |
| Other | 0.965 (0.825–1.128) | .6518440099 | 1.204 (1.014–1.431) | .0342304205 | 1.078 (0.796–1.460) | .6267388540 |
| Baseline extraintestinal manifestations | 0.796 (0.599–1.058) | .1163310799 | 1.138 (0.858–1.511) | .3694573968 | 0.823 (0.419–1.619) | .5734586315 |
| Baseline all-cause hospitalization |  |  |  |  |  |  |
| 0 | Reference | – | Reference | – | Reference | – |
| 1–2 | 1.044 (0.881–1.236) | .6206875126 | 1.114 (0.937–1.325) | .2217938493 | 1.308 (0.932–1.835) | .1207152839 |
| ≥ 3 | 1.190 (0.815–1.739) | .3679801716 | 1.171 (0.849–1.613) | .3355147024 | 0.919 (0.338–2.499) | .8685080023 |
| Baseline CCI score | 1.071 (0.986–1.163) | .1046698601 | 0.993 (0.908–1.086) | .8752279865 | 0.979 (0.868–1.103) | .7250586381 |
| Baseline mental disorder | 0.949 (0.778–1.159) | .6094999816 | 0.930 (0.761–1.136) | .4762176014 | 1.498 (0.972–2.310) | .0673073273 |
| Baseline conventional therapy duration, days |  |  |  |  |  |  |
| 0–90 | Reference | – | Reference | – | Reference | – |
| ≥ 91 | 1.067 (0.921–1.236) | .3894102561 | 1.060 (0.906–1.241) | .4679986013 | 1.077 (0.799–1.452) | .6243674038 |
| Index year |  |  |  |  |  |  |
| 2013 | Reference | – | Reference | – | Reference | – |
| 2014 | 1.034 (0.733–1.457) | .8501065501 | 1.936 (1.428–2.625) | .0000210366 | 0.289 (0.145–0.578) | .0004398802 |
| 2015 | 0.964 (0.695–1.336) | .8245293746 | 1.782 (1.329–2.388) | .0001121388 | 0.235 (0.136–0.406) | .0000002008 |
| 2016 | 0.995 (0.728–1.359) | .9743961231 | 2.225 (1.648–3.006) | .0000001834 | 0.441 (0.272–0.714) | .0008763037 |
| 2017 | 1.049 (0.757–1.454) | .7734212826 | 2.486 (1.793–3.446) | .0000000470 | 0.366 (0.225–0.597) | .0000567813 |
| 2018 | 1.204 (0.853–1.699) | .2919074059 | 3.683 (2.613–5.191) | < .0000000001 | 0.506 (0.323–0.794) | .0030400187 |
| 2019/2020 | 1.829 (1.271–2.631) | .0011361726 | 6.169 (4.241–8.973) | < .0000000001 | – | – |

Analyses were adjusted for each variable listed in the table.

Abbreviations: CCI, Charlson Comorbidity Index; CI, confidence interval.

## Supplementary Table 10. Cox proportional hazard model: Rate of switching or discontinuation of each individual second-line biologic in patients with ulcerative colitis.

| **Independent variables** | **Second-line biologic treatment** | | | | | | | |
| --- | --- | --- | --- | --- | --- | --- | --- | --- |
|  | **Adalimumab** | | **Infliximab** | | **Ustekinumab** | | **Vedolizumab** | |
|  | **Hazard ratio  (95% CI)** | ***P* value** | **Hazard ratio  (95% CI)** | ***P* value** | **Hazard ratio  (95% CI)** | ***P* value** | **Hazard ratio  (95% CI)** | ***P* value** |
| Age | 1.005  (0.996–1.015) | .2609128488 | 0.990  (0.983–0.998) | .0143584421 | 0.998  (0.966–1.031) | .8854504683 | 0.998  (0.992–1.004) | .5115285101 |
| Sex |  |  |  |  |  |  |  |  |
| Female | Reference | – | Reference | – | Reference | – | Reference | – |
| Male | 0.874  (0.681–1.121) | .2882870182 | 0.986  (0.782–1.243) | .9068028590 | 0.507  (0.180–1.425) | .1976150758 | 1.026  (0.845–1.246) | .7952454223 |
| Race/ethnicity |  |  |  |  |  |  |  |  |
| White or Caucasian | Reference | – | Reference | – | Reference | – | Reference | – |
| Black or African American, Asian, unknown/other | 0.681  (0.477–0.973) | .0350626011 | 0.721  (0.493–1.054) | .0915465453 | 0.305  (0.048–1.959) | .2108112584 | 0.836  (0.596–1.172) | .2980841033 |
| Body mass index, kg/m^2^ |  |  |  |  |  |  |  |  |
| Missing, < 18.5–24.9 | Reference | – | Reference | – | Reference | – | Reference | – |
| ≥ 25 | 0.969  (0.740–1.268) | .8159491237 | 1.050  (0.821–1.343) | .6987577365 | 1.216  (0.388–3.812) | .7367686730 | 0.948  (0.775–1.161) | .6070413355 |
| Baseline smoking | 0.979 (0.689–1.391) | .9048759767 | 0.975  (0.700–1.358) | .8831948783 | 0.624  (0.144–2.711) | .5290647870 | 0.856  (0.643–1.141) | .2895984445 |
| Baseline disease extent |  |  |  |  |  |  |  |  |
| Pancolitis | Reference | – | Reference | – | Reference | – | Reference | – |
| Other | 0.948  (0.718–1.250) | .7042004241 | 1.176  (0.924–1.497) | .1873605453 | 0.772  (0.260–2.288) | .6401326089 | 1.245  (1.017–1.524) | .0333204716 |
| Baseline extraintestinal manifestations | 0.984  (0.636–1.521) | .9416810141 | 1.089  (0.692–1.715) | .7116831552 | 1.384  (0.285–6.732) | .6870386522 | 0.991  (0.692–1.419) | .9614804938 |
| Baseline all-cause hospitalization |  |  |  |  |  |  |  |  |
| 0 | Reference | – | Reference | – | Reference | – | Reference | – |
| 1–2 | 1.195  (0.910–1.570) | .1999178829 | 1.137  (0.869–1.488) | .3501133606 | 1.383  (0.396–4.831) | .6110583670 | 1.150  (0.930–1.422) | .1960210387 |
| ≥ 3 | 1.691  (0.934–3.063) | .0829941300 | 0.937  (0.525–1.671) | .8251182956 | 4.487  (0.638–31.560) | .1314439126 | 1.158  (0.742–1.807) | .5187259252 |
| Baseline CCI score | 1.007  (0.892–1.136) | .9143557194 | 1.064  (0.959–1.180) | .2392421023 | 0.999  (0.573–1.741) | .9960729684 | 1.066  (0.964–1.179) | .2138173671 |
| Baseline mental disorder | 0.831  (0.607–1.137) | .2475899248 | 1.026  (0.742–1.418) | .8785862046 | 0.335  (0.071–1.575) | .1660372666 | 1.088  (0.839–1.412) | .5239391373 |
| Baseline conventional therapy duration, days |  |  |  |  |  |  |  |  |
| 0–90 | Reference | – | Reference | – | Reference | – | Reference | – |
| ≥ 91 | 1.040  (0.809–1.336) | .7602251809 | 0.775  (0.612–0.980) | .0332592719 | 1.125  (0.410–3.087) | .8187136931 | 0.925  (0.764–1.118) | .4182952184 |
| Index year |  |  |  |  |  |  |  |  |
| 2013 | Reference | – | Reference | – | Reference | – | Reference | – |
| 2014 | 1.030  (0.680–1.561) | .8877768203 | 1.944  (1.090–3.466) | .0242652383 | 2.915  (0.274–31.038) | .3753010615 | 1.171  (0.798–1.720) | .4193686863 |
| 2015 | 1.028  (0.679–1.555) | .8964726741 | 1.549  (0.888–2.700) | .1230764904 | 1.183  (0.147–9.535) | .8746754121 | 1.170  (0.810–1.690) | .4038548595 |
| 2016 | 1.208  (0.791–1.846) | .3821215834 | 1.951  (1.136–3.349) | .0154010590 | 0.629  (0.085–4.665) | .6499492645 | 1.222  (0.844–1.770) | .2878859309 |
| 2017 | 1.429  (0.916–2.230) | .1157246789 | 2.055  (1.171–3.606) | .0120771169 | 0.953  (0.137–6.645) | .9616523320 | 1.310  (0.887–1.933) | .1748523022 |
| 2018 | 1.062  (0.621–1.817) | .8253646288 | 1.731  (0.931–3.218) | .0826661518 | 0.553  (0.044–6.987) | .6471119084 | 1.097  (0.704–1.710) | .6831408535 |
| 2019/2020 | 1.326  (0.641–2.744) | .4473938633 | 1.729  (0.839–3.564) | .1375734993 | 0.375  (0.024–5.872) | .4848734588 | 1.268  (0.716–2.244) | .4159297731 |

Analyses were adjusted for each variable listed in the table.

Abbreviations: CCI, Charlson Comorbidity Index; CI, confidence interval.

## Supplementary Figure 1. ROTARY study design.


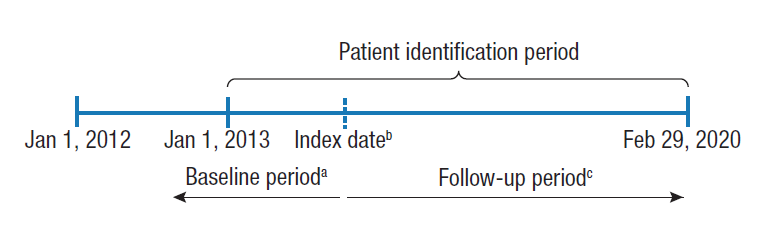


ROTARY, Real wOrld ouTcomes Across tReatment sequences in inflammatorY bowel disease patients. ^a^The baseline period was defined as the 12 months before the index date. ^b^The index date was defined as the first date of prescription or administration of a qualifying biologic (adalimumab, infliximab, vedolizumab, or ustekinumab) during the patient identification period. ^c^Follow up was defined as the period between the index date and whichever came first of the end of the second line of biologic treatment or the end of the study period.
